# The relationship between postoperative delirium and plasma amyloid beta oligomer

**DOI:** 10.1038/s41598-025-97577-0

**Published:** 2025-04-16

**Authors:** YoungSoon Yang, Ki Jin Jung, Yong Tae Kwak

**Affiliations:** 1 Department of Neurology, Cheonan Hospital, Soonchunhyang University College of Medicine, Cheonan, Korea; 2 Department of Orthopaedic Surgery, Cheonan Hospital Soonchunhyang University College of Medicine, Cheonan, Korea; 3 Department of Neurology, Hyoja Geriatric Hospital, Sanghari 33, Kuseong-myeon, Yongin-si, Kyeongki-do Korea

**Keywords:** Alzheimer’s disease, Amyloid beta oligomer, MDS-Oaβ, Postoperative delirium, Neuroscience, Cognitive neuroscience

## Abstract

Postoperative delirium (POD) is a frequent complication in older people undergoing general anesthesia surgery. We investigated the potential link between Alzheimer’s disease and POD by comparing plasma amyloid-beta oligomer levels (measured using the multimer detection system, MDS-OAβ) in patients who developed POD after general anesthesia surgery with those who did not. A total of 104 eligible participants were screened daily for delirium for three days postoperatively. After propensity score matching based on the ApoE4 allele, the final analysis included 31 patients with POD and 31 without POD. In the ICU, patients with delirium underwent immediate assessment with the Korean version of the Delirium Rating Scale-98 (K-DRS-98) and plasma MDS-OAβ levels. The control group (those without POD) received the same tests on the third postoperative day. Patients with POD had significantly higher MDS-OAβ values compared to those without POD. Within the POD group, MDS-OAβ values positively correlated with K-DRS-98 scores (both severity and total scores). These findings suggest an association between POD in older people undergoing general anesthesia surgery and elevated plasma amyloid oligomer levels. To definitively establish causality, further prospective studies are necessary.

## Introduction

With the recent rapid increase in life expectancy and advances in anesthesia techniques, the demand for general anesthesia in the older people undergoing surgery has increased significantly. Simultaneously, associated with this surge, there is a rapid increase in postoperative delirium(POD) among these patients, leading to delayed recovery and cognitive impairments, thereby causing an escalation in physical and social costs. While POD can occur from the first to the fifth day after surgery, it typically peaks between 2 and 3 days postoperatively, especially in older people^1–4^. In young individuals, the risk of POD is only 2–3%^1–4^. However, in older people, the risk increases significantly, ranging from 50–70%^5,6^. Furthermore, patients who experience POD have a significantly higher 30-day mortality rate, ranging from 7 to 10%, compared to only 1% in patients without POD^7,8^. Additionally, POD is known to increase the probability of severe functional impairment and transfer to care facilities by 2–3 times compared to patients without POD^9,10^. Despite extensive studies on the pathophysiology of POD, the precise pathophysiology remains unknown. Therefore, prevention and treatments for this condition are not yet established. Currently, the most effective preventive measures for postoperative delirium involve managing established risk factors such as fasting duration, choice of anesthesia, adequate fluid administration pre- and post-surgery, and proper blood pressure controls.

While delirium and dementia have distinct symptoms and causes, both preclinical and epidemiological evidence suggest a strong link between POD and dementia. Preclinical studies suggest an association between general anesthesia and the formation and aggregation amyloid-beta protein(Aβ) and amyloid-beta oligomerization^11^, with a particular emphasis on the correlation with inhalational anesthetics^12^. Additionally, delirium is considered an independent risk factor for dementia, and individuals with dementia are more likely to develop POD^13^. While the exact mechanism is unclear, epidemiological studies also suggest a possible connection between POD in older people and Alzheimer’s disease(AD).

To date, most studies exploring the link between POD and AD primarily relied on cerebrospinal fluid (CSF) biomarkers or amyloid PET scans. However, these methods are often cost-prohibitive and have limited availability, leading to a scarcity of studies with inconsistent findings. For instance, one study using amyloid PET scan found no association between cerebral amyloid burden and POD^14^, while another study observed an association with the severity of POD^15^. Recent technological advancements allow for measuring Aβ in plasma, prompting research into its potential link with POD. While existing plasma biomarkers like Aβ42, Aβ40, and their ratios effectively reflect amyloid plaques and aid in AD diagnosis, their ability to capture the dynamic, post-surgical changes in brain remains unclear. This parallels the limited clinical utility of amyloid PET scans, crucial for AD diagnosis but offering little beyond. Summarizing past research using CSF, plasma biomarkers, and amyloid PET scans to investigate the link between POD and AD, it appears there is no clear correlation between brain amyloid status and POD.

Aβ is formed from amyloid precursor proteins, and during this process, small soluble amyloid beta proteins known as amyloid-beta oligomers (AβOs) are generated. These AβOs are not only highly toxic to brain cells^16,17^, but are also associated with synaptic dysfunction, induction of tau pathology, neuroinflammation, impaired axonal transport, and neuronal death^18^. In addition to AD diagnosis, AβOs are closely associated with the presence, severity of symptoms, and progression of the disease^19^. It is known that AβOs may provide a more accurate diagnosis or better reflect progression of AD than Aβ plaque load itself or individual monomers like Aβ42 and Aβ40. Studies in AD patients consistently demonstrate that while plasma levels of Aβ42 and Aβ40 monomers decrease with disease progression, AβOs tend to increase until a certain stage. This occurs because monomers convert into oligomers, causing a decrease in monomer levels. Consequently, AβOs formed from these monomers are considered better biomarkers of the ongoing AD pathophysiology in the brain^20,21^. However, despite their recognized importance, quantitatively analyzing AβOs in blood has posed a significant challenge. The recently developed the multimer detection system (MDS-OAβ) method enables reliable measurement of plasma AβOs and is currently being used in clinical settings. Notably, the MDS-OAβ test shows not only high sensitivity for AD diagnosis but also associations with cognitive function^22,23^. Furthermore, studies have reported a significant correlation between MDS-OAβ levels and reduced volume in brain regions implicated in AD, suggesting its potential as a biomarker for predicting both diagnosis and progression of disease^24^.

In this study, we focus on postoperative delirium (POD), which occurs after surgical procedures under general anesthesia. Although patients were monitored in the intensive care unit (ICU) postoperatively, our interest is specifically in POD rather than delirium associated with prolonged ICU stays (ICU delirium). This distinction is important, as the pathophysiology and risk factors may differ between these two entities. By concentrating on POD in the surgical context, we aim to investigate factors specifically related to surgery and anesthesia in older people. Lastly, while patients were monitored in the intensive care unit (ICU) postoperatively, our focus is specifically on postoperative delirium (POD), which occurs after surgical procedures under general anesthesia, rather than delirium associated with prolonged ICU stays (ICU delirium). This distinction is important, as pathophysiology and risk factors may differ between these two entities. By concentrating on POD in the surgical context, this study investigates two primary objectives: first, to determine if there are differences in MDS-OAβ levels between patients aged ≥ 70 years with and without POD. Second, to assess the correlation between the severity of POD and MDS-OAβ values in patients who experience POD. By exploring these aims, we hope to shed light on the potential link between POD and amyloid beta status in the brain.

## Methods

### Ethics

This study received ethics committee approval and written informed consent from all participants (IRB #2023-02-038, Soonchunhyang University College of Medicine, Cheonan Hospital) and was conducted from February 2023 to April 2024. Written informed consent was obtained from each patient. All methods were performed in accordance with the relevant guidelines and regulations.

### Participants

This prospective cross-sectional study enrolled consecutive patients aged ≥ 70 years undergoing elective general anesthesia non-intracranial surgery (e.g., subtotal gastrectomy, cervical/lumbar laminectomy, or total hip/knee arthroplasty) between February 2023 and April 2024. Patients who were admitted to the surgical ICU for more than 24 h at Soonchunhyang Cheonan Hospital, affiliated with Soonchunhyang University, were included in this study. Patients who did not require ICU admission were excluded to maintain consistency in the monitoring environment.

Their global cognitive function was assessed using the Korean Mini-Mental State Examination (K-MMSE) on the day before surgery. To compare with patients experiencing POD, the control group included all participants aged ≥ 70 years who underwent similar surgeries with general anesthesia during the same period and did not develop POD during the study. The exclusion criteria for both study groups were as follows: (1) patients with the MMSE score < 24 or dementia due to various etiologies; (2) patients scheduled to undergo neurosurgery; (3) patients with daily tranquilizer or antipsychotic medication use and extensive alcohol consumption(more than 15 standard alcoholic drinks per week for men or more than 8 for women); (4) patients with known CNS diseases, such as psychiatric illnesses; (5) patients who could not complete the delirium testing, such as those who were expected to remain intubated postoperatively, particularly if they would be sedated for postoperative ventilation; (6) patients with hearing or visual deficits that would prevent neuropsychological testing. All study participants underwent routine blood tests and ApoE gene analysis. ApoE genotyping was conducted using the PCR-RFLP (Polymerase Chain Reaction-Restriction Fragment Length Polymorphism) method on preoperative blood samples, ensuring baseline measurements that were not affected by surgical or postoperative factors.

Patients with MMSE scores below 24 or diagnosed with dementia were excluded to maintain a study population with normal baseline cognitive function. Including individuals with pre-existing cognitive impairment or psychiatric illnesses could introduce confounding variables, as these conditions are associated with altered amyloid-beta levels and a higher baseline risk of POD. This exclusion helps ensure that the observed associations are more specifically related to POD in cognitively normal older people.

### Detection of delirium and clinical and neuropsychological evaluation

Although POD can occur even after four days post-surgery, our study focused on the first three postoperative days. Patients without POD often recover quickly and are discharged by day three, limiting data collection beyond this point. Additionally, aligning the sampling timeframe to within three days for both groups minimizes potential confounders from time-dependent changes in plasma amyloid beta oligomer levels, ensuring consistency in our measurements. Following surgery, all participants were assessed daily for delirium using the Korean Version of the Delirium Rating Scale-98 (K-DRS-98). If a patient exhibited signs of delirium, plasma MDS-OAβ measurement was conducted. POD was defined using the K-DRS-98, with severity scores of ≥ 18.5 or total scores of ≥ 20.5 indicating delirium, as established in a previous study^25^. The control group, consisting of patients who did not develop POD, underwent the same assessments on the third postoperative day.

### The measurement of oligomerization of Aβ in plasma

MDS-OAβ values were utilized for the measurement of oligomeric Aβ (OAβ)^26,27^. Venous blood was collected in 10-mL sodium-heparin-containing tubes and immediately centrifuged at 1,500 × g for 10 min. Aliquots of plasma samples were then stored at − 70 °C until analysis to ensure sample stability. This test was performed when delirium was suspected after surgery, while patients who did not show delirium undergo the tests third days postoperatively. Prior to the assay, plasma samples were thawed at 37 °C for 15 min. The multimer detection system is a modified ELISA in which PBR-1 (synthetic Aβ made by PeopleBio Inc.) was spiked into plasma, and the mixture was incubated at 37 °C for 48 h. The incubated plasma sample mixture and serially diluted standard samples were added to their respective wells, and the plates were incubated at room temperature for 1 h. Subsequently, 100 µL/well of an enhanced chemiluminescence substrate solution (Rockland Immunochemicals Inc., Limerick, PA, USA) was added, and the Relative Luminescence Unit (RLU) signal was detected using a Victor 3TM multi-spectrophotometer. Dilutions that provided signals within the linear range of the standard curves were employed for converting RLU values to determine the concentration of oligomerized Aβ.

### Statistical analysis

Descriptive statistics were presented as mean ± standard deviation (SD) for continuous data and as number and percentages for categorical data. Differences between patients with and without POD were assessed using chi-square tests for categorical variables and t-tests for continuous variables. Linear regression analyses were conducted to identify factors influencing the development of POD. Due to the non-randomized assignment of patients with and without POD, potential confounding variables could bias the results. Particularly, the interaction between the ApoE4 allele and plasma amyloid status necessitates further evaluation. To address this, we performed a propensity score-based matching for ApoE4 allele counts across POD and non-POD groups, ensuring comparable MDS-Oaβ values. Finally, we used Pearson correlation analysis to determine the correlation between MDS-Oaβ values and delirium severity in participant with POD. Statistical analyses were performed using SPSS version 24.0 (SPSS, Inc, Chicago, IL, USA).

## Results

### Characteristics of all study participants and the variables influencing MDS-Oaβ values in participants without POD

**Fig. 1:**
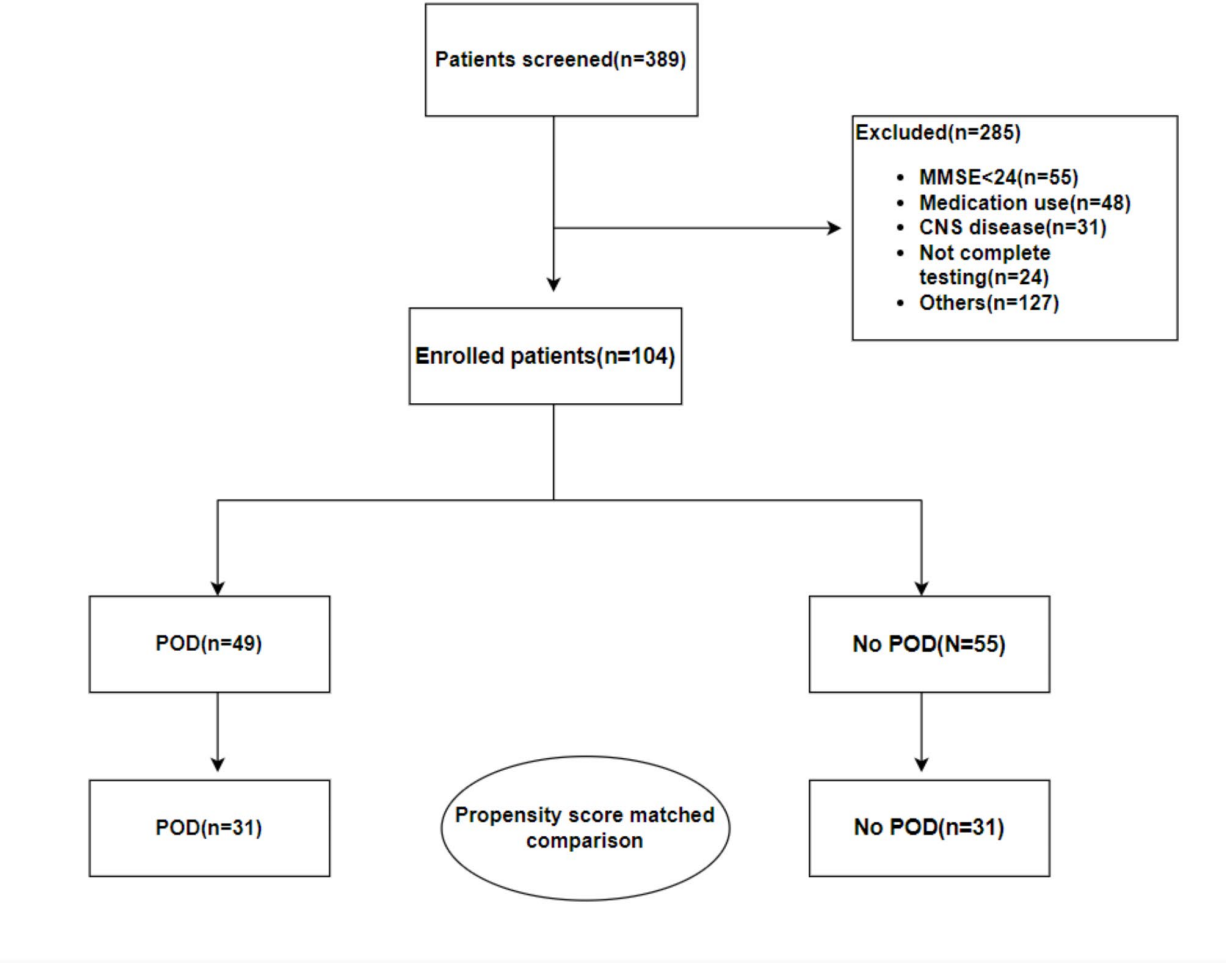
Flow chart of patient eligible for the study from the study population.

During the study period, a total of 1872 surgeries were conducted under general anesthesia, 389 of which involved patients aged 70 and older. After applying exclusion criteria (Fig. 1), 104 subjects were included as index cases for analysis (Table 1). There were significant differences in MDS-Oaβ values and the number of ApoE4 alleles between patients with and without POD (Table 1). The MDS-Oaβ assay used in this study has a lower limit of quantification (LLOQ) of 0.063 ng/mL and a reliable detection range of 0.313 to 10 ng/mL, as specified by the assay manufacturer, PeopleBio Inc. In our analysis, all plasma MDS-Oaβ concentrations fell within this detection range, ranging from 0.30 to 1.20 ng/mL, ensuring reliable measurements. Due to the significant impact of ApoE4 allele count on MDS-Oaβ values in patients without POD, as confirmed by regression analysis (Table 2), we conducted propensity score matching for the number of ApoE4 alleles (Fig. 1). Clinical characteristics of the study population before and after matching were described in Tables 1 and 3, respectively.

**Table 1:** Baseline characteristics.

Variables	Overall, *N* = 104	Delirium		*p*-value
		Yes, *N* = 49	No, *N* = 55	
Age, years	76.4 ± 5.9	76.6 ± 6.6	76.1 ± 5.3	0.642
Female gender (%)	56 (53.8%)	22 (44.9%)	34 (61.8%)	0.115
Education	8.1 ± 4.4	7.5 ± 4.5	8.7 ± 4.1	0.170
MMSE	26.0 ± 1.1	26.0 ± 1.3	26.0 ± 0.8	0.434
Number of ApoE4 gene	0.57 ± 0.72	0.84 ± 0.75	0.31 ± 0.60	0.000
Operating time (min)	221.4 ± 50.4	227.7 ± 54.1	215.9 ± 46.7	0.236
Surgery type, n (%)				
General	32	16	16	
Orthopedic	40	19	21	
Urological	11	1	10	
Vascular	21	13	8	
K-DRS-98 severity	13.3 ± 12.9	26.3 ± 4.8	1.7 ± 2.1	0.000
K-DRS-98 total	15.4 ± 14.4	30.1 ± 5.0	2.0 ± 2.9	0.000
Sampling time (h)	47.2 ± 29.8	18.5 ± 17.4	73.8 ± 3.9	0.000
MDS-Oaβ (ng/ml)	0.71 ± 0.25	0.89 ± 0.15	0.55 ± 0.21	0.000
Incidence of delirium(%)	47.1 (5)			

K-DRS-98; Korean Version of Delirium Rating Scale-98.

**Table 2:** Results of multiple linear regression analysis predicting MDS-Oaβ in patients without postoperative delirium(*n* = 55).

Variables	B	SE(B)	Beta	t	*p*-value
Age	0.006	0.005	0.147	1.201	0.235
Sex	0.084	0.047	0.192	1.784	0.091
Education	0.006	0.005	0.111	1.047	0.300
MMSE	− 0.035	0.031	− 0.136	− 1.136	0.262
Number of ApoE4 gene	0.248	0.037	0.702	6.646	0.000

**Table 3:** Baseline characteristics after propensity score matching.

Variables	Overall, *N* = 62	Delirium		*p*-value
		Yes, *N* = 31	No, *N* = 31	
Age, years	76.5 ± 5.9	77.0 ± 6.6	76.0 ± 5.2	0.471
Female gender (%)	32 (51.6%)	15 (48.4%)	17 (54.8%)	0.800
Education	8.2 ± 4.7	7.4 ± 4.8	8.9 ± 4.1	0.149
MMSE	26.0 ± 1.2	26.0 ± 1.4	26.0 ± 0.8	1.000
Number of ApoE4 gene	0.58 ± 0.50	0.58 ± 0.50	0.58 ± 0.50	1.000
Operating time(min)	218.9 ± 49.6	222.1 ± 52.5	216.2 ± 47.5	0.610
Surgery type, n(%)				
General	20	10	10	0.157
Orthopedic	25	13	12	
Urological	7	1	6	
Vascular	10	7	3	
K-DRS-98 severity	13.2 ± 12.9	26.5 ± 4.8	1.7 ± 2.7	0.000
K-DRS-98 total	15.5 ± 14.6	30.6 ± 4.8	2.5 ± 2.9	0.000
Sampling time	47.8 ± 29.8	19.0 ± 18.3	72.8 ± 2.9	0.000
MDS-Oaβ(ng/ml)	0.72 ± 0.23	0.85 ± 0.16	0.59 ± 0.22	0.000

K-DRS-98; Korean Version of Delirium Rating Scale-Revised-98.

### The variables related with POD

**Fig. 2:**
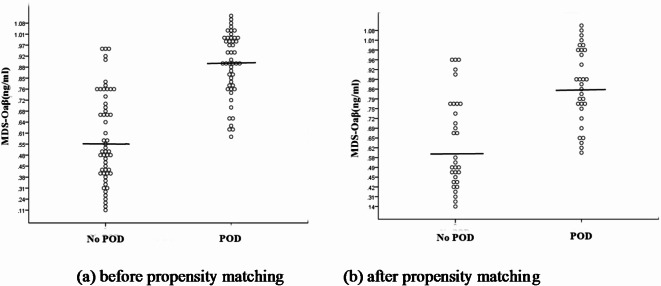
Plasma MDS-Oaβ (ng/ml) values for patients without POD and with POD. Scatter plot presented mean with range. Before and after propensity matching for Apo E4 allele number, significant difference was observed in plasma MDS-Oaβ value between patients with POD and without(*P* < 0.001). POD; postoperative delirium.

After propensity score matching, the study included 31 patients with POD and 31 without POD. MDS-Oaβ values were significantly higher in patients with POD compared to those without (*p* < 0.0001) (Fig. 2). No significant differences were found in age, sex, education, preoperative MMSE score, surgery type, or other variables between the two groups.

### The relationship between the MDS-Oaβ values and severity of delirium in patients with POD

**Fig. 3:**
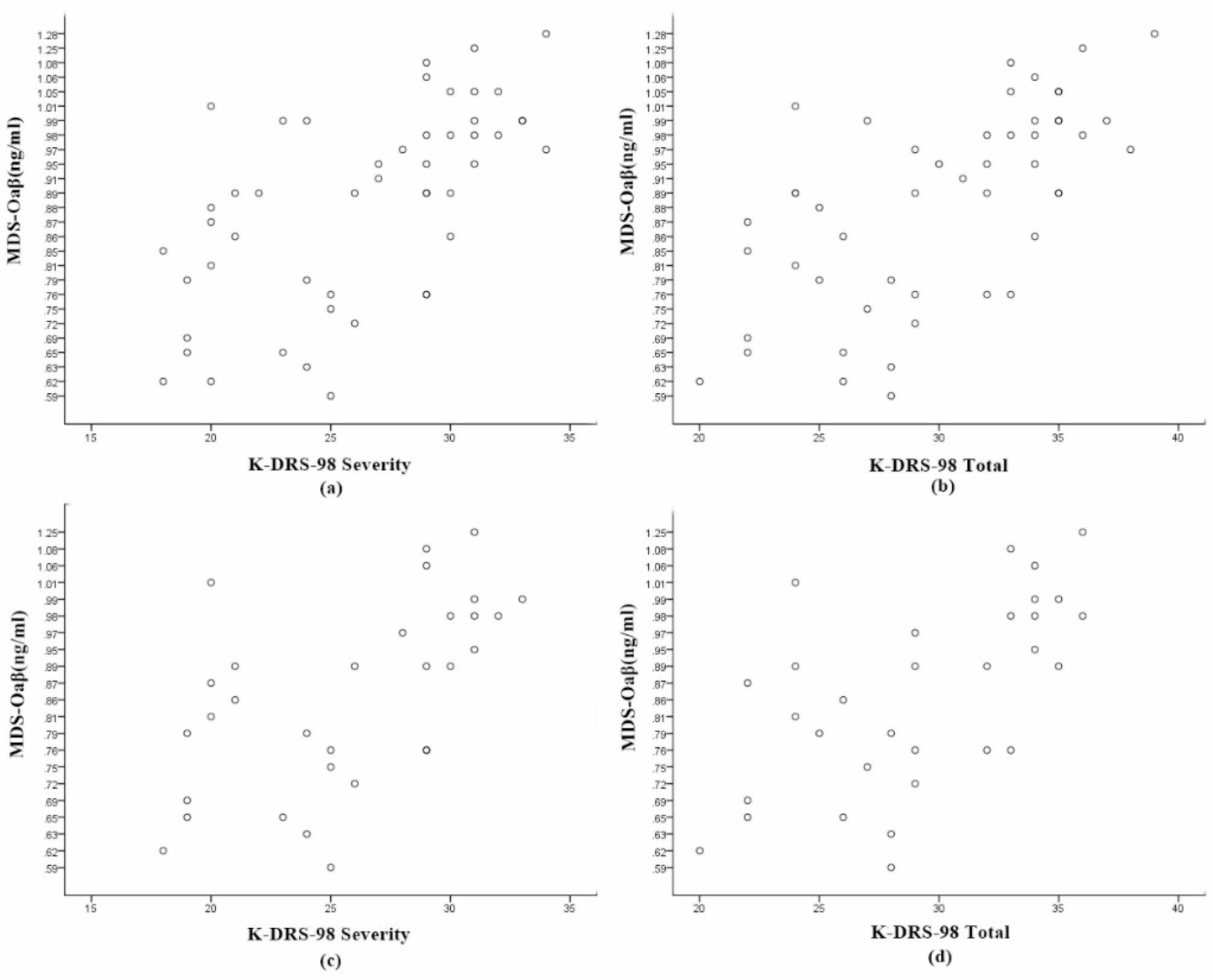
Scatterplot of the value of MDS-Oaβ and K-DRS-98 severity (A), K-DRS-Total (B), K-DRS-98 severity after propensity matching (c), K-DRS-Total after propensity matching (d). Both before and after propensity matching, the correlation between MDS-Oaβ and K-DRS-98 severity, as well as between MDS-Oaβ and K-DRS-Total, has a p-value below 0.001.

Within the patients with POD, MDS-Oaβ values showed a positive correlation with both K-DRS-98 severity (*r* = 0.58, *p* < 0.01) and K-DRS-98 total scores (*r* = 0.62, *p* < 0.01). This significant correlation was also observed before propensity score matching (Fig. 3).

## Discussion

Recently, MDS-Oaβ values have been utilized as a biomarker reflecting the pathophysiology of AD. This study compared MDS-Oaβ values between patients with and those without POD, in patients aged ≥ 70 years who underwent general anesthesia surgery. The research findings revealed a significantly higher MDS-Oaβ value in patients with POD compared to those without. Additionally, there was a significant positive correlation between the MDS-Oaβ value and the severity of delirium in patients with POD. Considering the epidemiological findings that POD in older people is associated with an increased risk of AD, it is highly likely that POD is linked to the pathophysiology of AD in this group. Therefore, to further explore this possibility, numerous studies have investigated the connection between POD and AD biomarkers.

According to a recent meta-analysis, there is a negative correlation between the levels of Aβ42 in CSF and the occurrence of POD. Conversely, no significant associations were found with other CSF biomarkers: Aβ40, total tau (T-tau), phosphorylated tau (P-tau), and the Aβ42/T-tau ratio^28^. Studies investigating plasma Aβ or the Aβ40/42 ratio have largely reported non-significant results regarding their association with POD^29–32^. Recently, Payne et al.^29^ examined the relationship between plasma Aβ levels and Aβ ratio (AβR) in 100 older people, both before and after surgery, to determine their association with POD. They observed a postoperative increase in AβR compared to preoperative levels in surgical patients. However, this rise in AβR, or in individual plasma Aβ values, did not correlate significantly with the frequency or severity of delirium. The authors concluded that their findings do not support a connection between plasma amyloid and POD^29,33^. In contrast to these studies, our findings revealed significantly higher MDS-Oaβ values in patients with POD compared to those without POD. Furthermore, a significant positive correlation was observed between MDS-Oaβ values and delirium severity in patients with POD, suggesting a clear link between amyloid pathology and POD. However, since our correlation analysis did not adjust for confounding factors, these results should be interpreted with caution. These results differed from previous findings, prompting the question: what factors could underlie this discrepancy?

Previous studies investigating the relationship between amyloid-beta levels and POD have reported inconsistent findings, especially when measuring monomeric forms of amyloid-beta in plasma and CSF. In AD patients, CSF levels of Aβ42 are typically decreased due to its deposition in amyloid plaques, while plasma levels can be variable and influenced by peripheral factors, making them less reliable indicators of central amyloid pathology. In delirium patients, studies measuring plasma Aβ40, Aβ42, and their ratios have often found no significant association with POD occurrence or severity^29–32^. These inconsistencies may stem from the use of monomeric amyloid-beta forms biomarker, which may not sensitively reflect acute changes in amyloid metabolism associated with delirium. The key difference between our study and previous ones lies in the selection of the amyloid biomarker. Unlike prior studies that employed amyloid monomers like plasma Aβ40 and Aβ42, we utilized the MDS-Oaβ test to specifically evaluate amyloid beta oligomers (AβOs). Initially recognized as intermediate substances in amyloid plaque formation, AβOs are now understood to be more neurotoxic than other amyloid peptides. Studies have shown that elevated levels of AβOs are associated with cognitive impairment in Alzheimer’s disease and other neurodegenerative conditions, highlighting their significant role in cognitive dysfunction. Additionally, unlike static markers such as amyloid plaques, AβOs exhibit phasic behavior, varying with disease progression or state. Our finding of higher MDS-Oaβ levels in patients with POD aligns with these observations, suggesting that AβOs may contribute to the pathophysiology of POD. This biomarker has the potential to manifest in the pathophysiology of AD from a very early stage, potentially earlier than other conventionally used biomarkers^35^.

Studies have shown that ApoE purified from AD patient brains enhances Aβ oligomerization in an isoform-dependent manner, with the strongest effect observed for the E4 isoform. In particular, individuals with two copies of the E4 isoform form more brain Aβ than E3 homozygotes^36^ and be cleared more slowly from the body^37^. A study tracking long-term cerebrospinal fluid changes before the transition from normal cognition to mild cognitive impairment (MCI) in ApoE4 carriers revealed a lower Aβ42/Aβ40 ratio compared to non-carriers, suggesting amyloidopathy prior to symptom onset^38^. Our study showed significantly higher MDS-Oaβ values and an increased number of ApoE4 alleles in patients with POD. While regression analysis revealed no significant association between the number of ApoE4 alleles and POD occurrence, it did significantly influence MDS-Oaβ values in patients without POD. These findings suggest that the ApoE4 allele may have indirectly influenced the results of this study. Therefore, this study presents two sets of results: one from the overall study population and the other from a propensity score matching analysis conducted after reconstructing the study population based on the ApoE4 allele.

The observed elevation in MDS-Oβ values among patients with POD compared to those without POD in this study suggests two potential explanations. Firstly, POD delirium might arise from a post-surgical increase in Aβ, potentially triggering pathophysiological processes like neuroinflammation or synaptic dysfunction, ultimately contributing to the delirium pathology. This hypothesis finds support in the established ability of commonly used inhaled anesthetics, such as isoflurane, to promote Aβ oligomerization and accumulation in both in vitro and in vivo models. Isoflurane induces a time-dependent increase in Aβ oligomerization and accumulation in the brain. Moreover, evidence suggests that various anesthetics can alter amyloid metabolism through diverse pathways, leading to amyloidopathy. These findings raise concerns about the potential acceleration of AD progression in individuals exposed to these agents^39^. The second possibility is that even in patients who appear clinically normal, if they already have long-standing Aβ plaques (indicating ongoing AD pathology), the elevated Aβ levels during the surgical process could accelerate silent brain pathology. This acceleration could contribute to the development of both delirium and increase the risk of AD onset. However, due to the cross-sectional design of our study, it is not possible to determine which possibility is more likely. It merely indicates an association between POD and AβOs in some way. A prospective study, measuring MDS-Oβ values before surgery and comparing those with and without POD afterwards, is needed to definitively understand the relationship between MDS-Oβ values and POD.

This study has several limitations. First, we were unable to adjust for potential confounding factors such as comorbidities, type and depth of anesthesia, duration of surgery, and degree of blood loss. Due to limitations in data availability and the relatively small sample size, it was not feasible to include these variables in our analysis. While we attempted to control for some confounders through propensity score matching based on the ApoE4 allele, we acknowledge that other factors may have influenced our findings. Second, the impact of general anesthetic surgery on MDS-Oaβ values in older people aged 70 and above remains unclear. If, hypothetically, MDS-Oaβ values increase immediately after surgery and gradually decrease over time, the difference in sampling times could potentially influence the results. Patients with POD had immediate assessments at delirium onset, while the control group was assessed three days after surgery (Tables 1 and 3). This difference in sampling timing could potentially influence the results. Third, our study is the heterogeneity of the surgical procedures included. The varying types of surgeries and anesthesia methods could influence the incidence of POD and plasma amyloid-beta levels. Fourth, due to delirium’s fluctuating nature, some cases may have been missed if participants were asymptomatic during assessment, potentially leading to an underestimation of its true incidence, Lastly, as previously mentioned, the cross-sectional design precludes establishing causation between delirium and AβOs.

This study, however, revealed a statistically significant association between POD and amyloid status. Among patients aged 70 or above undergoing general anesthesia surgery, those with POD exhibited significantly higher MDS-Oaβ values compared to those without delirium. Additionally, a positive correlation was found between MDS-Oaβ values and delirium severity, suggesting a link between elevated AβOs and the severity of delirium. To our knowledge, this is the first study to investigate MDS-Oaβ in relation to POD. The limited research on this biomarker in the context of acute cognitive disturbances, such as POD, restricts our ability to directly compare findings. Future research with larger and prospective cohorts, including studies utilizing preoperative MDS-Oaβ measurements, is necessary to confirm these findings and explore potential preventive or therapeutic strategies for POD based on these observations.

## Data Availability

Anonymized data from this study are available for academic purposes upon reasonable request. Enquiries can be directed to the corresponding author.
